# Epidemiology of dengue fever in Guatemala

**DOI:** 10.1371/journal.pntd.0008535

**Published:** 2020-08-19

**Authors:** Leticia del Carmen Castillo Signor, Thomas Edwards, Luis E. Escobar, Yolanda Mencos, Agnes Matope, Mariana Castaneda-Guzman, Emily R. Adams, Luis E. Cuevas

**Affiliations:** 1 Ministerio de Salud Publica y Asistencia Social de Guatemala, Guatemala City, Guatemala; 2 Centre for Drugs and Diagnostics Research, Liverpool School of Tropical Medicine, Liverpool, United Kingdom; 3 Department of Fish and Wildlife Conservation, Virginia Tech, Blacksburg, VA, United States of America; 4 Tropical Clinical Trials Unit. Liverpool School of Tropical Medicine, Liverpool, United Kingdom; Institute for Disease Modeling, UNITED STATES

## Abstract

Dengue fever occurs worldwide and about 1% of cases progress to severe haemorrhage and shock. Dengue is endemic in Guatemala and its surveillance system could document long term trends. We analysed 17 years of country-wide dengue surveillance data in Guatemala to describe epidemiological trends from 2000 to 2016.Data from the national dengue surveillance database were analysed to describe dengue serotype frequency, seasonality, and outbreaks. We used Poisson regression models to compare the number of cases each year with subsequent years and to estimate incidence ratios within serotype adjusted by age and gender. 91,554 samples were tested. Dengue was confirmed by RT-qPCR, culture or NS1-ELISA in 7097 (7.8%) cases and was IgM ELISA-positive in 19,290 (21.1%) cases. DENV1, DENV2, DENV3, and DENV4 were detected in 2218 (39.5%), 2580 (45.9%), 591 (10.5%), and 230 (4.1%) cases. DENV1 and DENV2 were the predominant serotypes, but all serotypes caused epidemics. The largest outbreak occurred in 2010 with 1080 DENV2 cases reported. The incidence was higher among adults during epidemic years, with significant increases in 2005, 2007, and 2013 DENV1 outbreaks, the 2010 DENV2 and 2003 DENV3 outbreaks. Adults had a lower incidence immediately after epidemics, which is likely linked to increased immunity.

## Author summary

Dengue is the most common mosquito-borne virus, and a major cause of fever, with an estimated 390 million infections annually. Guatemala, in Central America, has had ongoing dengue transmission since the 1990s. Its national surveillance system monitors outbreaks and seasonal trends of infections to inform public health responses. We have analysed 17 years of surveillance data collected from 2000 to 2016, to describe seasonal trends, outbreak years, and the fluctuating prevalence of the four dengue serotypes. Laboratory data from 91,554 individual serum samples were included, of which 7.8% were positive for dengue. All four dengue serotypes circulate in the country, with dengue 1 and 2 being the predominant serotypes. This is important, as it increases the likelihood of dengue infections being followed by a new infection with a different serotype, which can lead to severe dengue. We also report that adults in Guatemala have a lower likelihood of infection the year after an epidemic, which might be linked to an increased immunity in the population.

## Introduction

Dengue is widespread across the tropics with up to 3.97 billion individuals living in areas at risk of transmission [1], with an estimated 390 million infections occurring each year [2]. Its extensive geographical range and incidence caused an estimated 1.14 million disability adjusted life years lost in 2013 alone [3] at an economic cost of at least USD 8.9 billion [4].

The number of cases reported worldwide each year has increased from 15,497 cases in the 1960s, to one million in the 2000s [5] and 96 million cases per year in the last decade [2]. These increases are due to parallel increases in urbanization, travel and population density, particularly in the Americas and South-East Asia, facilitating dengue’s geographical expansion [6] outside traditional endemic areas [7] [8] [9].

The four dengue virus (DENV) serotypes (DENV 1 to 4) share 65–70% of their genomic sequence [10] and overlap spatially and temporally [11], with changing yearly patterns and fluctuating levels of serotype-specific immunity [12]. Infection with one serotype provides serotype-specific immunity without significant cross protection for other serotypes and a follow-on infection with a different DENV serotype carries an increased risk of disease severity through antibody mediated enhancement [13].

Surveillance of the incidence of DENV serotypes and outbreaks is essential to forecast the risk of disease burden and to enable reactive measures such as vector control and social mobilisation [14]. Moreover, regionally emerging arboviruses, particularly Zika and Chikungunya [15], have complicated dengue diagnosis and surveillance, as symptoms and geographic ranges are similar and serological diagnostics of flaviviruses cross react [16].

Dengue had a low incidence and was not considered a public health problem in Guatemala during most of the twentieth century, possibly as a by-product of the national malaria and yellow fever eradication campaigns [17]. However dengue viruses were reported since at least 1978, and all four serotypes had been reported by the 1990s [18] with a major outbreak of DENV3 with over 300,000 cases occurring in 1995 [18]. Systematic descriptions of the incidence of all serotypes in recent decades, however, are missing despite increasing number of cases reported by health services. We have thus undertaken an analysis of 17-years of dengue surveillance data in Guatemala, to describe longitudinal trends from 2000 to 2016, and the emergence and overlap of DENV serotypes in the country.

## Methods

### Ethics statement

This analysis used secondary sources of data collected for surveillance purposes and ethical approval was not required. No experimental work was undertaken outside of the routine diagnostic tests and all data were anonymised prior to analysis.

### National surveillance

Surveillance of arboviral infections in Guatemala is conducted by the National Epidemiology Centre and the National Public Health Laboratories (Centro Nacional de Epidemiologia and Laboratorio Nacional de Salud, LNS) of the Ministry of Health. The surveillance network comprises 340 primary care centres and 44 hospitals situated in the 29 health areas of the country. Surveillance is based on the regular reporting of data by the health facilities and is mostly passive, with some time-limited active surveillance during outbreaks. Health facilities submit serum samples to the LNS for laboratory confirmation of clinical cases, with samples sent in ice boxes via road transport. Test requests include patient information such as gender, age, address, history of travel, hospitalization, date of illness onset, and symptoms. The LNS test all samples received during endemic months, but only a proportion of samples during epidemic periods, when the laboratory prioritises severe and hospitalised cases. The laboratory has the capacity to test for dengue, chikungunya and Zika infections, with the aim of testing samples for all three viruses, except during outbreaks, when the most likely causative infection is prioritised. The LNS reports surveillance data to the National Epidemiology Centre, which in turn advises the Ministry of Health. Reports of new outbreaks, new arboviruses, and serious cases are in turn reported to the Pan American Health Organisation, along with monthly reports of passive surveillance data.

The LNS routinely holds surveillance records of all samples received from 2000 to 2016 for national and international reporting. We performed descriptive analyses on the data available to detect spatial and temporal patterns of the cases reported by LNS across the study period. Information regarding location of the cases and the health centres was available for 2016, and this information was used to compare the origin of the cases versus the health centre visited to determine patterns of displacement to receive health services.

### Diagnostic algorithms and case definitions

Dengue case definitions have changed with the advent of new diagnostics and updates of international case definitions. Between 2000 and 2010, testing for DENV was based on culture in clone C6/36 cells of *Aedes albopictus*, with confirmation based on immunofluorescent staining and fluorescence microscopy. In 2009 the LNS introduced the NS1 enzyme-linked immunosorbent assays (ELISA) to detect DENV antigens in serum, and since 2010 testing of acute cases has utilised the Centers for Disease Control (CDC) DENV 1–4 RT-qPCR assay that serotypes the virus with a limit of detection of between 5–10 copies [19]. A further assay that simultaneously detects DENV, Chikungunya and Zika (Trioplex), became available in 2016 [20]. However, the CDC 1–4 RT-qPCR continues to be used to confirm and serotype all Trioplex-positive samples, as this test had not been validated at the time of its introduction. Samples in the surveillance database with positive results to any of these tests are recorded as “confirmed dengue case”. Since 2000, samples from patients with symptoms over 5 days have also been tested with IgM antibody capture ELISA (MAC**-**ELISA) [21], and samples positive solely by this assay are reported here as “probable cases”.

## Results

**Fig 1:**
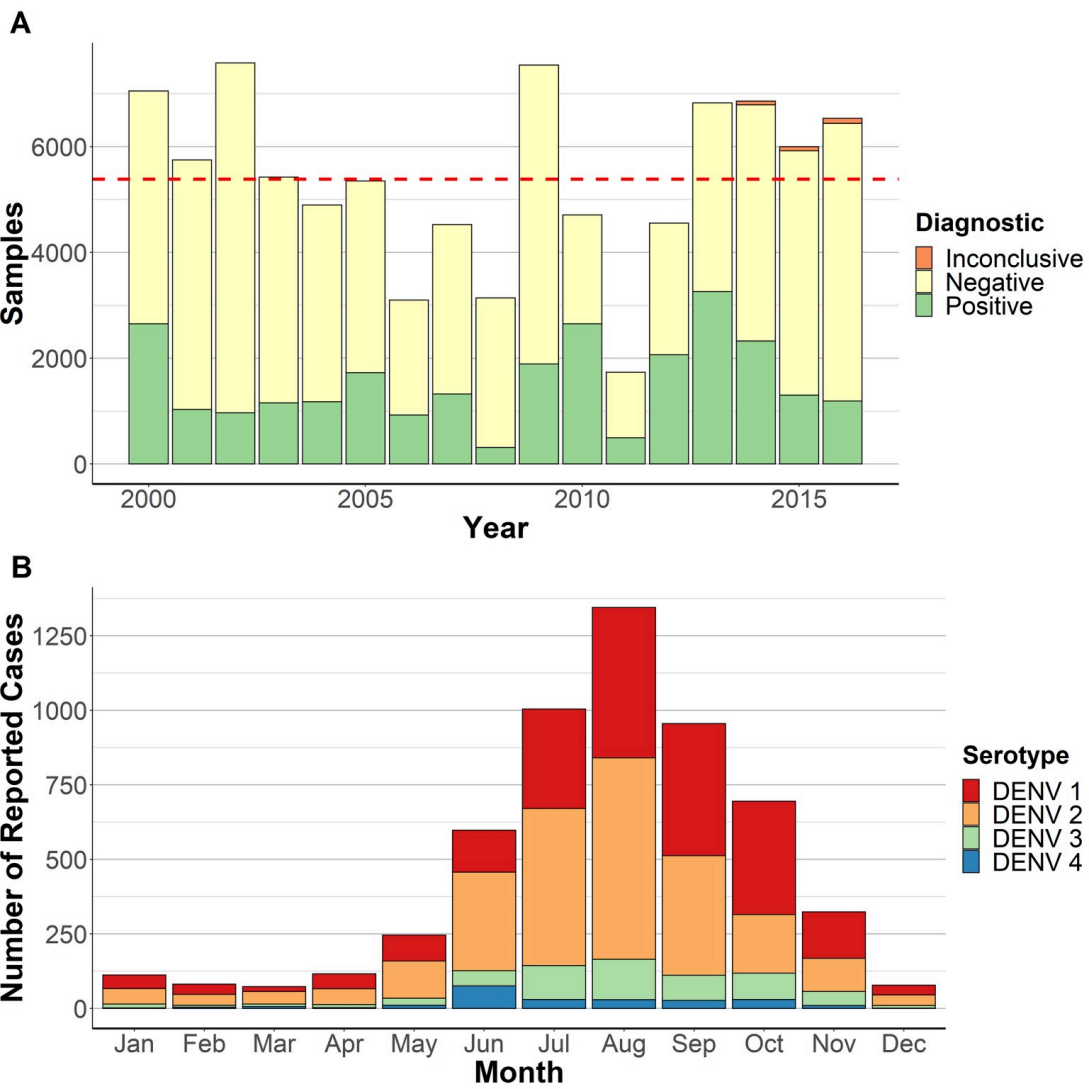
Temporal distribution of dengue in Guatemala. **A.** Annual number of samples received for dengue screening. The dashed line indicates the mean number of samples submitted across the study period (~5,300). **B**. Cumulative number of monthly cases of dengue serotypes 1, 2, 3, and 4.

**Fig 2:**
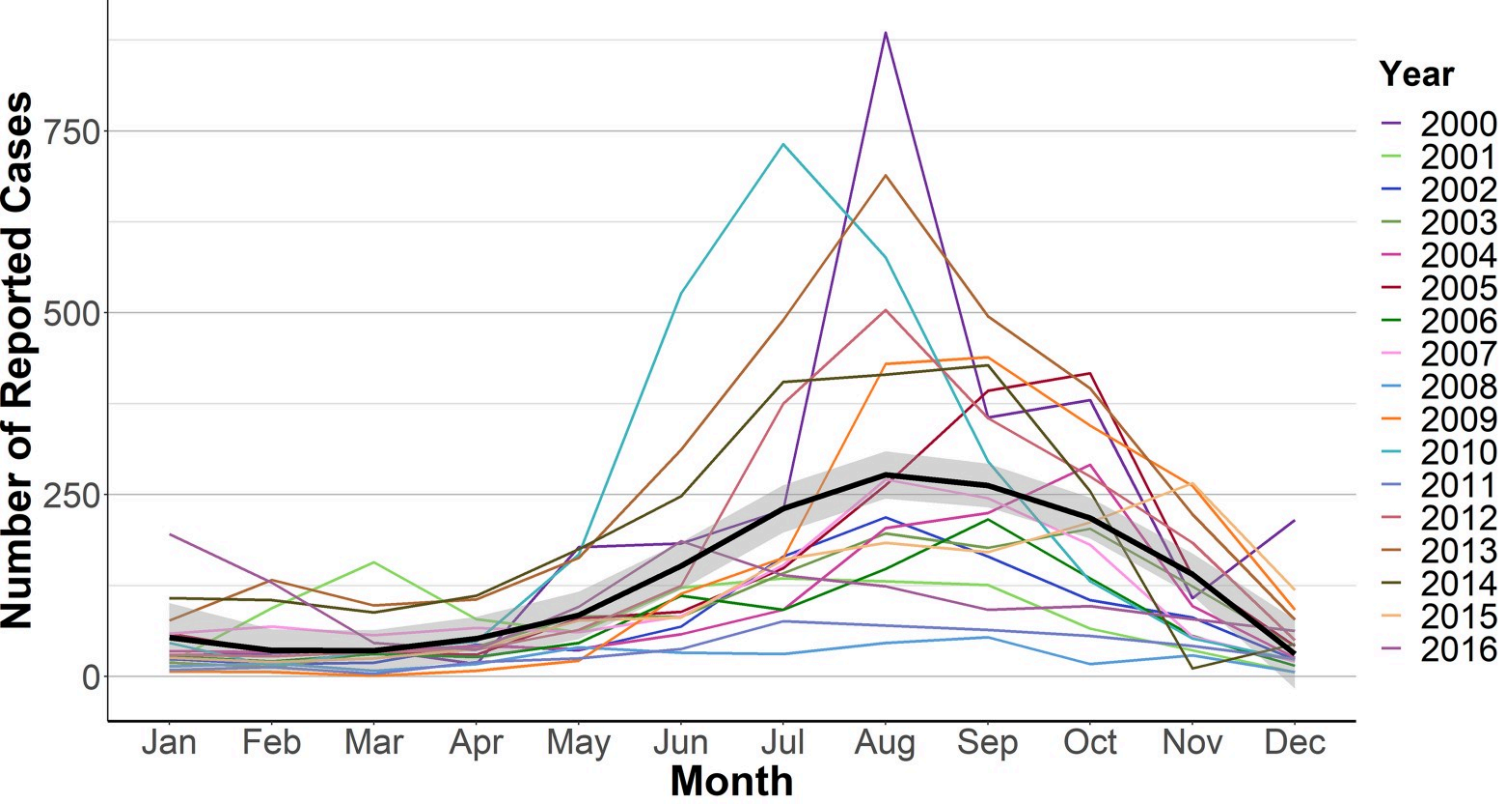
Monthly distribution of dengue cases in Guatemala (2000–2016). The mean annual number of cases is shown by a black line and the shaded areas describe the 95% confidence interval.

A total of 91,554 samples were received by the LNS from 2000 to 2016. The number of samples received varied over the years (Fig 1). Dengue infection was confirmed by RT-qPCR, viral culture or NS1 ELISA in 7097 (7.8%) samples and a further 19,290 (21.1%) were positive by IgM ELISA (probable cases). Samples were more likely to be confirmed after the introduction of RT-qPCR in 2009. The number of probable cases was higher between 2005 and 2007 and 2012–2014, which were years preceded by periods of high dengue incidence (S1 Fig). Most cases occurred in the second half of the year, with the majority occurring between July and October and a peak incidence in August (Figs 1 and 2 and S2 Table). Notable exceptions to this pattern were the outbreaks of DENV2 in 2010, which was detected in May, and DENV4 in 2001, with 94% of cases recorded in June (S3 Fig). The DENV3 outbreak in 2003, DENV1/DENV3 outbreak in 2004 and DENV1 outbreak in 2005 also had the highest case detection in October.

**Fig 3:**
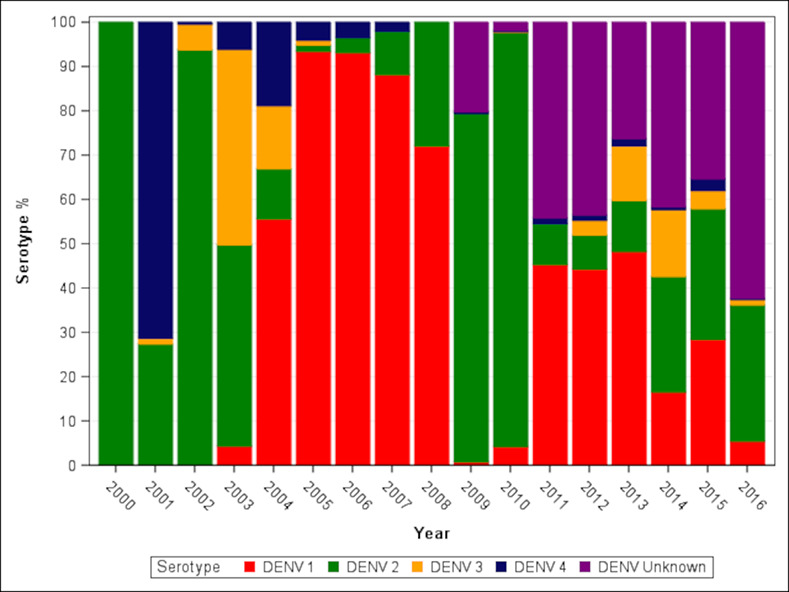
Annual percentage of confirmed dengue cases by serotype. Cases detected by NS1 ELISA but not serotyped are designated DENV unknown.

Serotyping by culture or RT-qPCR was available for 5619 (70.2%) of 7097 confirmed cases. DENV1, DENV2, DENV3, and DENV4 were detected in 2218 (39.5%), 2580 (45.9%), 591 (10.5%) and 230 (4.1%) cases, respectively. DENV1 and DENV2 had the highest frequencies, often over consecutive years (Figs 2 and 3). Peaks of DENV2 were observed in 2002 (262 cases) and 2003, followed by low numbers between 2006 and 2008 and a resurgence in 2009, when it caused the largest outbreak of the study period (1080 cases). DENV2 was often replaced by DENV1, most notably after the 2003 and 2010 epidemics. DENV1 predominated between 2004 and 2008, with peaks in 2005 and 2011–2015, causing 103 cases in 2011, 392 in 2012 and 542 in 2013 (S2 Fig). DENV3 caused outbreaks in 2003 and 2004 and in 2013 and 2014, when it circulated together with DENV1 and DENV2. DENV4 was the main serotype in 2001 and 2004 but was detected sporadically in other years.

**Fig 4:**
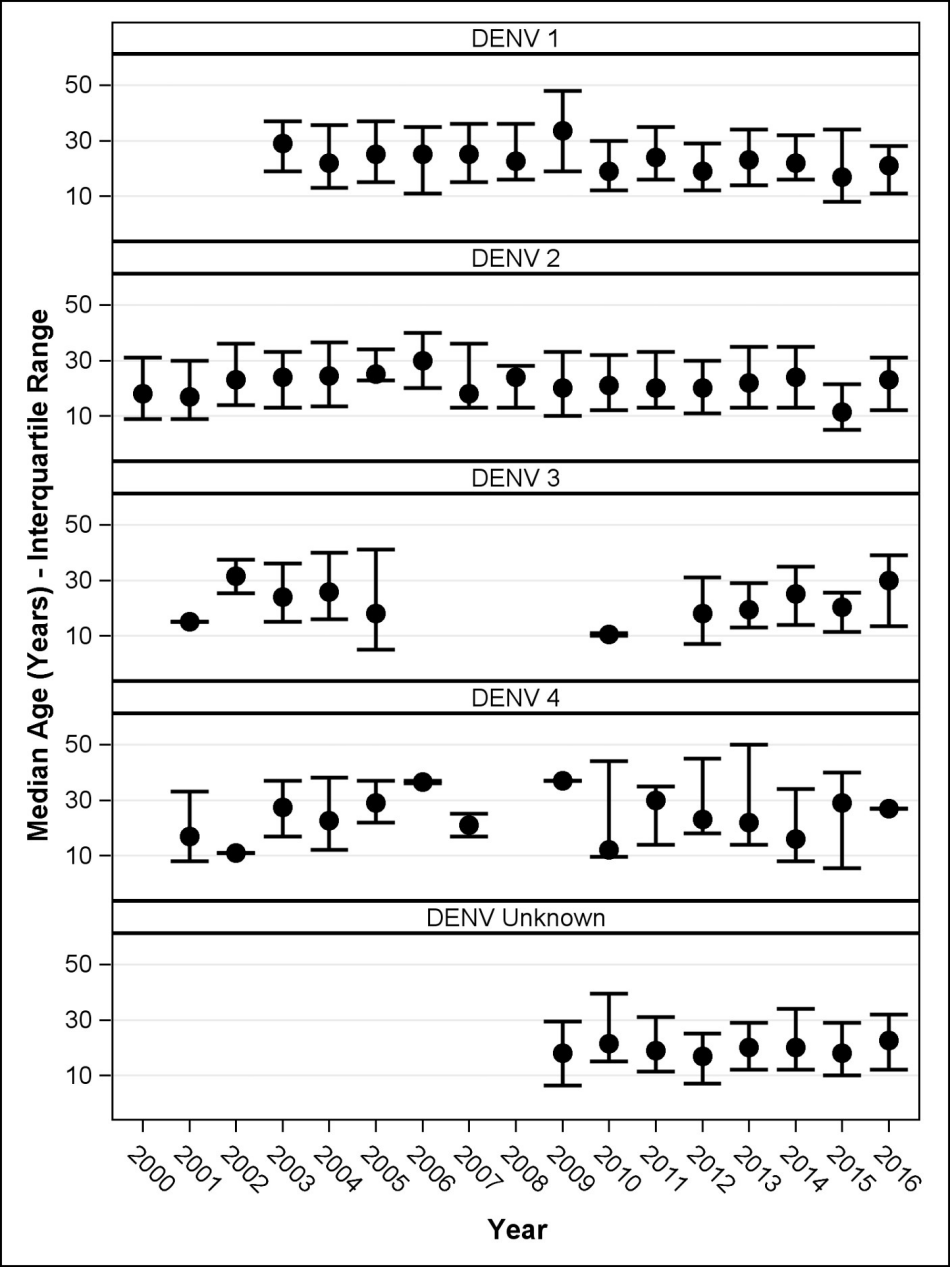
Median age of dengue cases per year. Error bars indicate interquartile range.

Dengue affected populations of all ages, with an average of 24.4 years and 18% of cases occurring in infants. The mean age of the cases varied over the years ranging from 19.1 to 26.5 years (Fig 4). However, during outbreaks the proportion of cases occurring among 5–20 and 20–60 age groups (2005, 2007 and 2013 DENV1, 2010 DENV2 and 2003 DENV3) was higher than in the following years, when the incidence was similar across all age groups (Table 1; S1 Table). Overall, 52.9% of all dengue cases occurred in women. Although some years affected higher proportions of men or women, there was no significant association with epidemics years or the predominance of a dengue serotype (S4 Fig).

**Table 1:** Incidence ratio of cases in each age group in 2010, the year with the major DENV 2 epidemic, and in 2011.

Age Group Cases—Comparisons between epidemic year and subsequent year (DENV 2 versus all DENV)		
Age Group	Epidemic year vs subsequent year	Incidence Ratio (95% CI*), p-value
<1	2010 vs. 2011	2.13 (0.14;31.86), 1.0000
1–5	2010 vs. 2011	2.01 (0.89;4.50), 0.1299
5–20	2010 vs. 2011	1.61 (1.18;2.20), 0.0003 **
20–60	2010 vs. 2011	2.23 (1.67;2.97), <0.0001 **
>60	2010 vs. 2011	1.93 (0.57;6.46), 0.8034

^*^ Confidence Interval

^**^ Significant, DENV: Dengue virus.

Some Departments (districts) provided health services to neighbouring Departments. For example, Departments receiving samples from other Departments included Guatemala, Santa Rosa, Quetzaltenango, and Zacapa. The Department of Guatemala received most imported patients from Jutiapa, Escuintla, El Progreso, and Santa Rosa. The Department of Santa Rosa analysed samples from Jutiapa, Guatemala, Chimaltenango, and Jalapa. The Department of Quetzaltenango analysed samples from the neighbouring Department of San Marcos and the Department of Zacapa analysed samples from El Progreso and Chiquimula.

**Fig 5:**
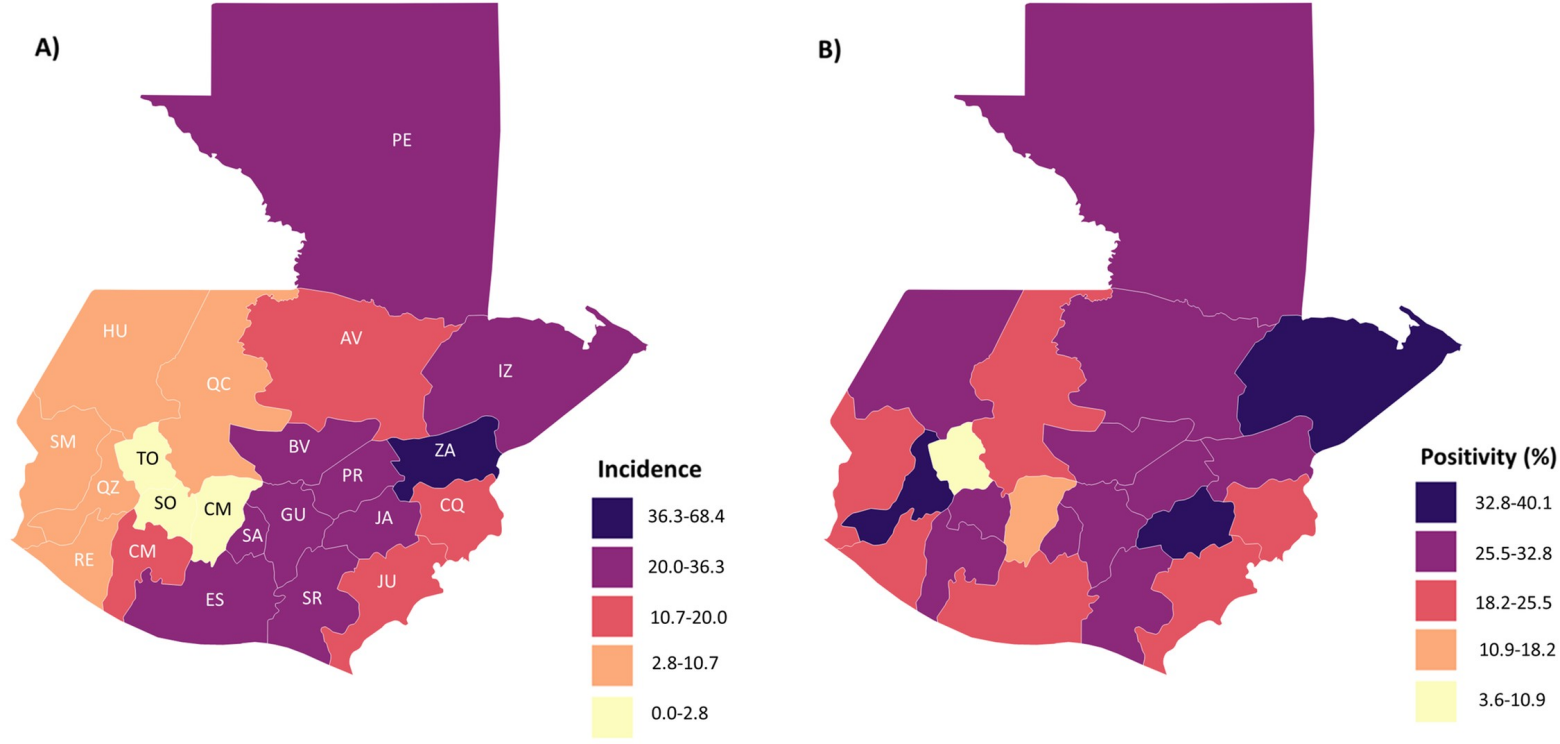
Geographic distribution of dengue in Guatemala (2000–2016). **A.** Number of dengue cases by Department (incidence per × 10,000 inhabitants). **B.** Proportion of samples tested that are positive for dengue. Darker colours denote higher values. (Population data from Wikipedia: Departments of Guatemala, 2011 population estimate).

Overall, the Department of Guatemala reported the highest number of cases followed by the Departments in the East of the country (i.e., Izabal, Zacapa, Santa Rosa, and El Progreso) (Fig 5), with the incidence by 10,000 population being highest in Zacapa. In contrast, the Departments in the Central Highlands of the country, such as Sololá, Chimaltenango, Retalhuleu, Sacatepéquez, and Huehuetenango had low numbers, reporting less than 100 cases.Quetzaltenango, Jalapa and Izabal had low incidence, but a high proportion of samples were dengue-positive (Fig 5).

## Discussion

Central America has a considerable burden of dengue, with an estimated incidence of 169 cases per 100,000 inhabitants, compared with 33.8 in the Caribbean, and 586.3 cases per 100,000 inhabitants in South America [22]. Dengue is hyperendemic, and seasonal in Guatemala with simultaneous circulation of all DENV serotypes [23]. In Guatemala, most infections were caused by DENV1 and DENV2, followed by DENV3 and DENV4, which mirrors reports from other Pan American Health Organization regions. The co-circulation of all four DENV serotypes increases the risk of second infections [24] with a different serotype, which leads to a higher risk of severe dengue [25]. Whilst previous DENV exposure can lead to the development of severe symptoms with the second infection; there is also some evidence of serological cross protection over the ensuing one to three years [26], which potentially may account for the reduction in the number of cases after epidemic years.

Epidemics for all dengue serotypes followed the same general pattern, with annual dengue cases peaking during the summer and autumn, between July and September, with some exceptions. This pattern was consistent among all serotypes. Interestingly the later peaks in dengue case detection between 2003 and 2005 occurred around two El Niño events [27], which have been shown to increase dengue transmission in Central America [28]. The understanding of the seasonality of dengue is an important epidemiological feature that enables timely control efforts for maximize prevention effectiveness, and public health planning [29].

Globally, DENV1 has been the most common serotype detected since 2010, particularly in Africa, Europe and the Western Pacific region [30]. However, DENV2 is the most commonly identified serotype in the Americas, and these two serotypes combined account for most cases in Guatemala. The infrequent detection of DENV4 is also mirrored from other settings, with monotypic DENV4 being responsible for only four percent of outbreaks globally between 2010 and 2016 [30].

The large 2010 DENV2 epidemic in Guatemala coincided with simultaneous DENV2 outbreaks across other Central and South American countries, with over 1.6 million reported cases [17]. Neighbouring Honduras, for example, reported 66,814 cases, with 92.5% them due to DENV2 [17]. The 2010 epidemic was followed by a lower incidence in the 5–20 and 20–60 age groups. The increased incidence in these age groups during epidemic years had been reported during DENV1 and DENV2 outbreaks in Singapore [31]. The reduced incidence in older age groups immediately after an epidemic [26] is thought to reflect herd immunity afforded by previous infection with the same serotype. Although it is widely believed that infection by a serotype confers lifelong immunity thanks to the development of serotype specific heterologous neutralising antibodies, recent studies describing homotypic re-infections in the short and long term have challenged this concept [32]^,^[33]. This suggests mechanisms facilitating viral evasion of the host immune system, escaping recognition or inhibiting the production of an antiviral state [34].

A large proportion of the samples tested here were dengue-negative, which was notably high in 2015 and 2016, coinciding with the large chikungunya [35] and zika [36] epidemics in the country. These diseases have a similar, unspecific clinical presentation [15]. Other common causes of fever compatible with dengue in the Latin American region include leptospirosis, typhoid fever, influenza, Rickettsiosis and less common arboviruses such as Oropouche and Mayaro [37, 38], which are likely to be investigated as potential dengue cases in surveillance laboratories. Testing samples using syndromic panels that include a wider range of pathogens is currently too costly and labour intensive for routine implementation. However, co-infections and parallel epidemics are well documented. For example, dengue and chikungunya co-circulated in Guatemala, with a report indicating that both viruses were present in 32% of samples in 2015 [35]. Thus, testing samples with more comprehensive diagnostic panels could generate broader information on the co-circulation of pathogens and lead to improved multi-disease surveillance. The development of multiplex diagnostics such as the CDC Trioplex [20] has simplified testing algorithms and improved detection of acute co-infections. However, serological tests still struggle to differentiate between DENV and Zika virus infections [39], which may have inflated the number of probable dengue cases in our analysis.

Passive surveillance is the standard approach used for dengue surveillance worldwide. This is due, in part, to the cost of implementing active case detection strategies for a disease with no specific treatment and limited vaccine uptake. Passive surveillance for dengue in Latin America has underestimated the number of cases by a factor between 3.5 and 19 [14], and as a result, the number of cases presented here is likely to underestimate the real burden of dengue in Guatemala. This underreporting can lead to the underappreciation of the impact of dengue in a region, leading to insufficient public health efforts to control transmission [40]. The reliance on passive surveillance can also result in an over-representation of severe cases that require the clinic and hospital services, adding bias to epidemiological data, particularly regarding disease severity and patient outcomes. It has been estimated that ambulatory patients, who make up the majority of cases in Latin America, incur the biggest economic cost [41], and that these patients are often particularly under-represented by passive surveillance. Whilst most costs to hospitalised patients are direct healthcare costs, the effect of dengue on non-hospitalised patients incurs a large socio-economic costs, mainly due to reduced productivity [42], which drives the overall cost of dengue to PAHO countries over US$ 3 billion [42].

The surveillance of dengue in Guatemala, and other countries in Latin America, would benefit from studies to optimise thresholds of excess reporting, which could trigger outbreak responses [43], and the initiation of active surveillance. Data from Australia, one of the few high income countries with dengue transmission which utilises active surveillance, has shown that rapid notification and case finding enables the minimisation of transmission by triggering public health responses [44]. However, active surveillance systems are expensive and implementation would require a robust cost-benefit analysis [45].

A limitation of the geographical analysis presented here is that the selection of sentinel sites and reporting centres reflects prior knowledge of areas considered to be endemic. Therefore, our analysis represents a combination of purposely selected centres that historically have reported more cases, which in turn resemble the health service referral patterns of patients and health seeking behaviour; together with a higher incidence of dengue in specific geographical areas. Our data therefore describes the long term trends of dengue, but does not provide reliable data of its prevalence across geographical areas. A further limitation of our data is that diagnostic tests have changed over the years. These changes modify the proportion of tests that are positive, particularly the implementation of RT-qPCR in 2010, which is more sensitive than the cell culture-based methods it replaced, and are more efficient to process samples, increasing testing capacity and allowing screening a larger numbers of cases. Therefore the proportion of positive results would have varied over the years due to changes in tests performance and the varied case definitions used to select the samples for screening.

Dengue is the most prevalent mosquito-transmitted infection worldwide and causes important economic and human costs [45]. The analysis of countrywide surveillance data can provide insights into the present and past epidemiology of the disease at a country level [31, 46], and is essential for the direction of future control and surveillance efforts. Such data are particularly important for future dengue vaccine roll out, and the understanding of the potential effect of new interventions.

## Supporting information

S1 Table Incidence ratio of cases in each age group in epidemic years and subsequent years, stratified by dengue serotype. (DOCX)

S2 Table Thresholds of the endemic cycle of dengue in Guatemala. Month: month of the year. Cases expected: best fit model resembling the average numbers of monthly cases during the period 2000–2016. Minimum expected: lower limit of values from the confidence intervals of the model. Maximum expected: higher limit of values from the confidence intervals of the model; Maximum expected denotes the threshold to declare an epidemic in a specific month. Error: standard error. Note that these values allow declaration of an epidemic during any month of the year at the country level when Maximum expected cases are above the higher limit.
(DOCX)

S1 Fig Percentage of dengue positive, probable and negative cases per year. (TIF)

S2 Fig Number of confirmed dengue cases by month and serotype. Cases detected by NS1 ELISA but not serotyped are designated DENV unknown
(TIF)

S3 Fig Seasonality of dengue cases each year, stratified by serotype. (TIF)

S4 Fig Proportion of male cases per serotype per year. (TIF)
