# Supplementary material for: Epidemiology of dengue fever in Guatemala
Source: PLoS Negl Trop Dis. 2020 Aug 19;14(8):e0008535. doi: 10.1371/journal.pntd.0008535 (PMC7458341; doi:10.1371/journal.pntd.0008535)
Supplement: S1 Table — (DOCX) [file pntd.0008535.s001.docx]

**Supplementary tables**

**Supplement Table S1.** Incidence ratio of cases in each age group in epidemic years and subsequent years, stratified by dengue serotype.

| **Comparison of age groups in epidemic year and subsequent year by dengue type** | | | |
| --- | --- | --- | --- |
| **Dengue type** | **Age Group** | **Epidemic year versus subsequent year** | **Incidence Ratio (95% CI), P-value** |
| DENV 1 | <1 | 2012 vs 2013 | 3.39 (0.45;25.46), 1.0000 |
|  |  | 2013 vs 2014 | 2.07 (0.12;35.94), 1.0000 |
|  | 1-5 | 2005 vs 2006 | 3.12 (0.43;22.78), 1.0000 |
|  |  | 2006 vs 2007 | 0.54 (0.06;4.90), 1.0000 |
|  |  | 2007 vs 2008 | 1.01 (0.08;12.95), 1.0000 |
|  |  | 2012 vs 2013 | 1.90 (0.65;5.52), 1.0000 |
|  |  | 2013 vs 2014 | 7.41 (0.70;78.61), 0.2378 |
|  | 5-20 | 2005 vs 2006 | 5.26 (2.51;11.04), <0.0001 * |
|  |  | 2006 vs 2007 | 0.57 (0.25;1.32), 1.0000 |
|  |  | 2007 vs 2008 | 1.97 (0.79;4.87), 0.6307 |
|  |  | 2012 vs 2013 | 1.15 (0.84;1.57), 1.0000 |
|  |  | 2013 vs 2014 | 3.96 (2.46;6.38), <0.0001 * |
|  | 20-60 | 2005 vs 2006 | 4.31 (2.38;7.81), <0.0001 * |
|  |  | 2006 vs 2007 | 0.63 (0.33;1.22), 0.9951 |
|  |  | 2007 vs 2008 | 2.14 (1.04;4.36), 0.0225 * |
|  |  | 2012 vs 2013 | 1.02 (0.76;1.37), 1.0000 |
|  |  | 2013 vs 2014 | 4.49 (2.93;6.88), <0.0001 * |
|  | >60 | 2005 vs 2006 | 3.09 (0.12;78.45), 1.0000 |
|  |  | 2006 vs 2007 | 0.89 (0.02;40.83), 1.0000 |
|  |  | 2007 vs 2008 | 1.76 (0.04;80.92), 1.0000 |
|  |  | 2012 vs 2013 | 0.64 (0.10;4.07), 1.0000 |
|  |  | 2013 vs 2014 | 1.93 (0.25;15.06), 1.0000 |
| DENV 2 | 1-5 | 2010 vs 2011 | 11.00 (0.47;257.00), 0.5468 |
|  | 5-20 | 2010 vs 2011 | 14.02 (5.16;38.09), <0.0001 * |
|  | 20-60 | 2010 vs 2011 | 22.06 (8.14;59.77), <0.0001 * |
| DENV 3 | <1 | 2013 vs 2014 | 2.68 (0.09;82.18), 1.0000 |
|  | 1-5 | 2003 vs 2004 | 1.40 (0.17;11.26), 1.0000 |
|  |  | 2013 vs 2014 | 2.82 (0.34;23.40), 1.0000 |
|  | 5-20 | 2003 vs 2004 | 4.76 (1.97;11.53), <0.0001 * |
|  |  | 2013 vs 2014 | 1.40 (0.75;2.61), 1.0000 |
|  | 20-60 | 2003 vs 2004 | 3.77 (2.02;7.03), <0.0001 * |
|  |  | 2013 vs 2014 | 0.90 (0.52;1.57), 1.0000 |
|  | >60 | 2003 vs 2004 | 1.19 (0.03;44.03), 1.0000 |
|  |  | 2013 vs 2014 | 2.00 (0.05;73.78), 1.0000 |
| DENV 4 | 5-20 | 2001 vs 2002 | 21.26 (0.88;514.55), 0.0734 |
|  |  | 2004 vs 2005 | 7.37 (1.39;39.16), 0.0040 * |
|  | 20-60 | 2004 vs 2005 | 2.37 (0.86;6.50), 0.2295 |
|  | >60 | 2004 vs 2005 | 1.91 (0.05;70.45), 1.0000 |
