# Supplementary material for: Epidemiology of dengue fever in Guatemala
Source: PLoS Negl Trop Dis. 2020 Aug 19;14(8):e0008535. doi: 10.1371/journal.pntd.0008535 (PMC7458341; doi:10.1371/journal.pntd.0008535)
Supplement: S2 Table — Month: month of the year. Cases expected: best fit model resembling the average numbers of monthly cases during the period 2000–2016. Minimum expected: lower limit of values from the confidence intervals of the model. Maximum expected: higher limit of values from the confidence intervals of the model; Maximum expected denotes the threshold to declare an epidemic in a specific month. Error: standard error. Note that these values allow declaration of an epidemic during any month of the year at the country level when Maximum expected cases are above the higher limit. (DOCX) [file pntd.0008535.s002.docx]

**Supplementary Table S2. Thresholds of the endemic cycle of dengue in Guatemala.** Month: month of the year. Cases expected: best fit model resembling the average numbers of monthly cases during the period 2000-2016. Minimum expected: lower limit of values from the confidence intervals of the model. Maximum expected: higher limit of values from the confidence intervals of the model; Maximum expected denotes the threshold to declare an epidemic in a specific month. Error: standard error. Note that these values allow declaration of an epidemic during any month of the year at the country level when Maximum expected cases are above the higher limit.

| **Month** | **Cases expected** | **Minimum expected** | **Maximum expected** | **Error** |
| --- | --- | --- | --- | --- |
| January | 53.51114 | 6.240595 | 100.78168 | 23.97131 |
| February | 36.1139 | 7.130502 | 65.0973 | 14.69774 |
| March | 35.6101 | 7.64772 | 63.57247 | 14.17997 |
| April | 52.21956 | 22.22033 | 82.21878 | 15.21288 |
| May | 84.33858 | 51.94012 | 116.73705 | 16.42955 |
| June | 152.3065 | 119.90804 | 184.70497 | 16.42955 |
| July | 230.78792 | 198.38945 | 263.18638 | 16.42955 |
| August | 277.25888 | 244.86041 | 309.65734 | 16.42955 |
| September | 262.56756 | 232.56833 | 292.56678 | 15.21288 |
| October | 217.88383 | 189.92146 | 245.84621 | 14.17997 |
| November | 140.40138 | 111.41798 | 169.38478 | 14.69774 |
| December | 31.02638 | -16.25801 | 78.31077 | 23.97833 |
