# Supplementary figures and images for: Epidemiology of dengue fever in Guatemala
Source: PLoS Negl Trop Dis. 2020 Aug 19;14(8):e0008535. doi: 10.1371/journal.pntd.0008535 (PMC7458341; doi:10.1371/journal.pntd.0008535)

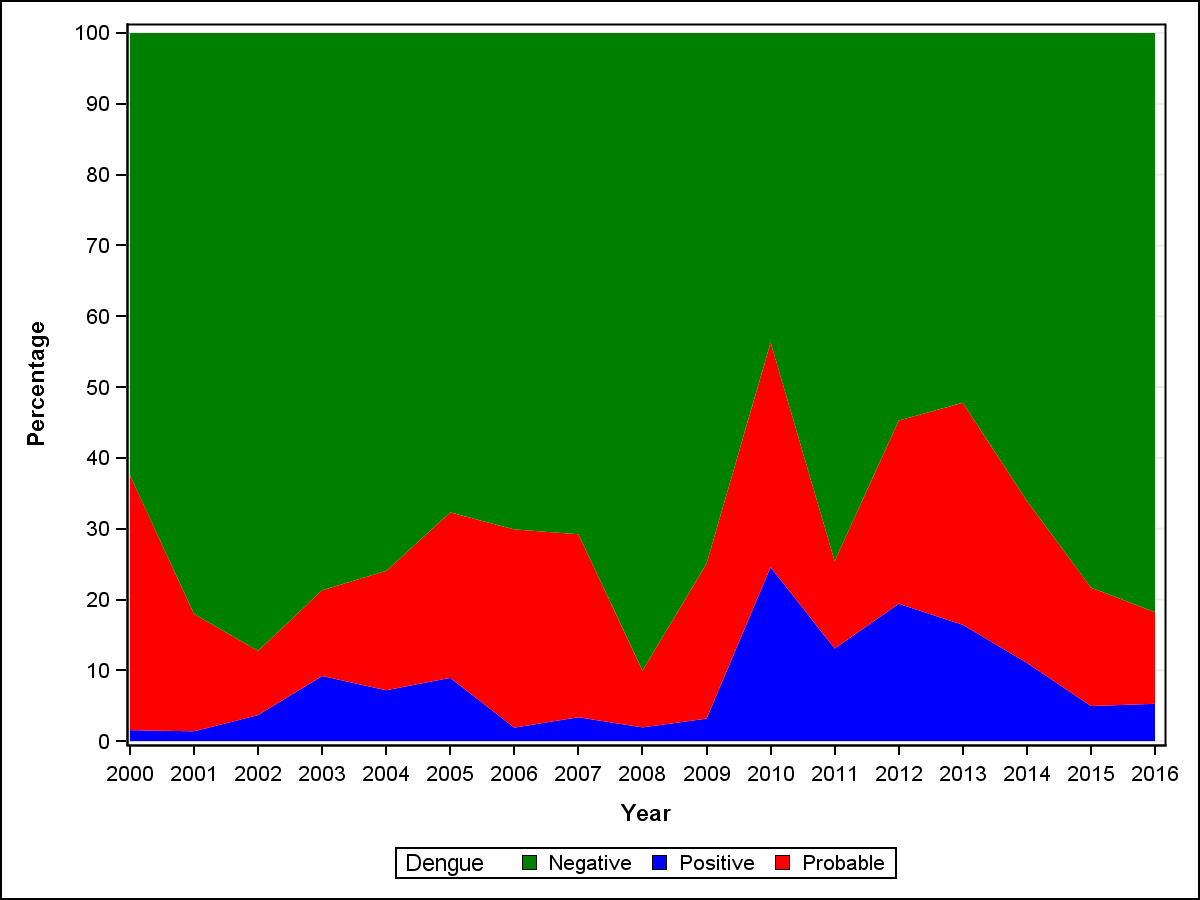

Supplement: S1 Fig — (TIF) [file pntd.0008535.s003.tif]

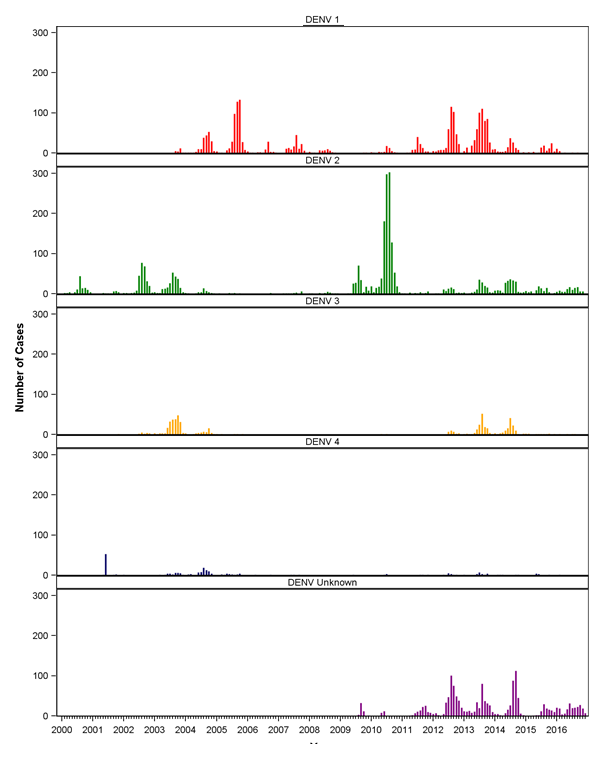

Supplement: S2 Fig — Cases detected by NS1 ELISA but not serotyped are designated DENV unknown (TIF) [file pntd.0008535.s004.tif]

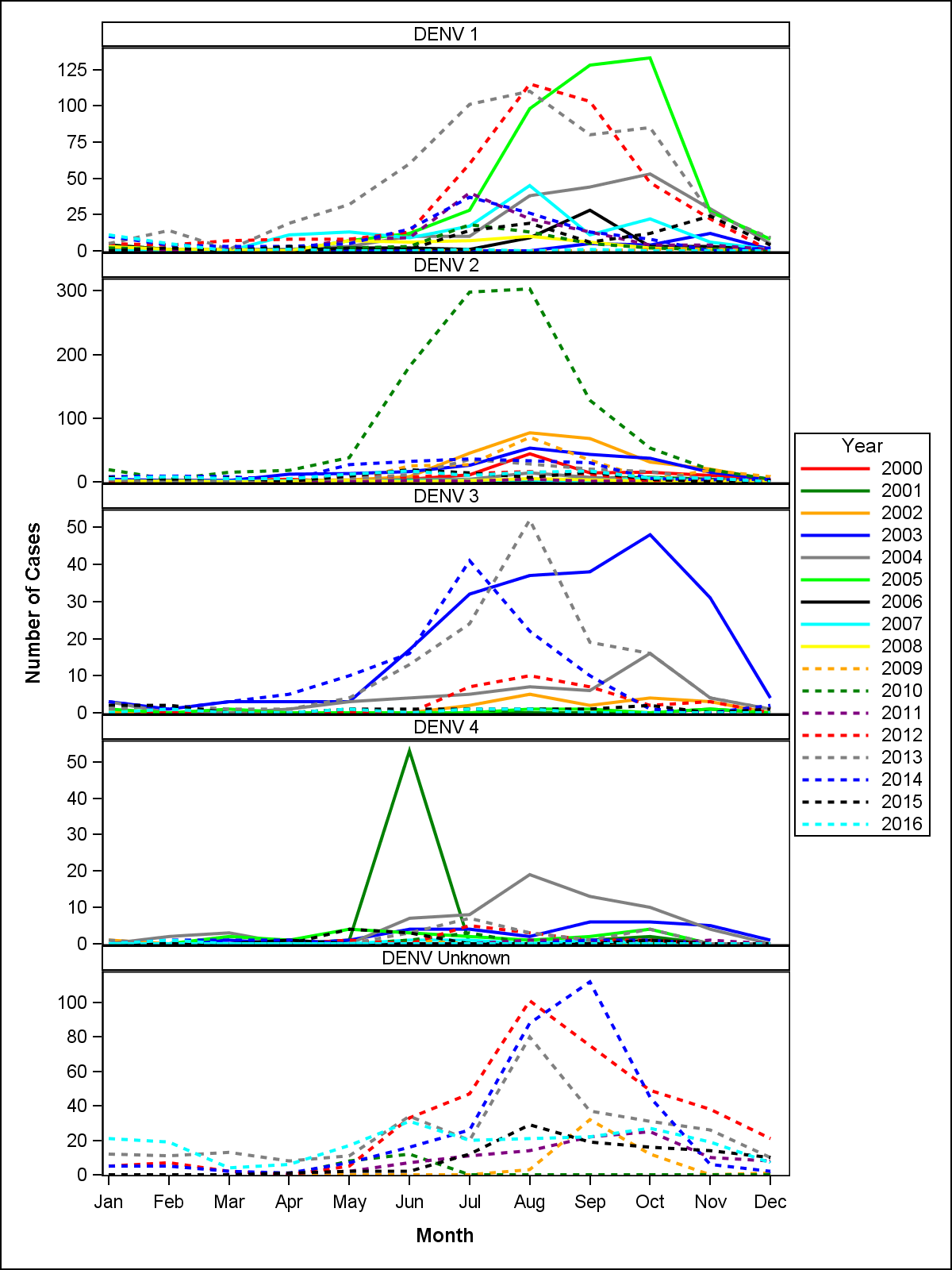

Supplement: S3 Fig — (TIF) [file pntd.0008535.s005.tif]

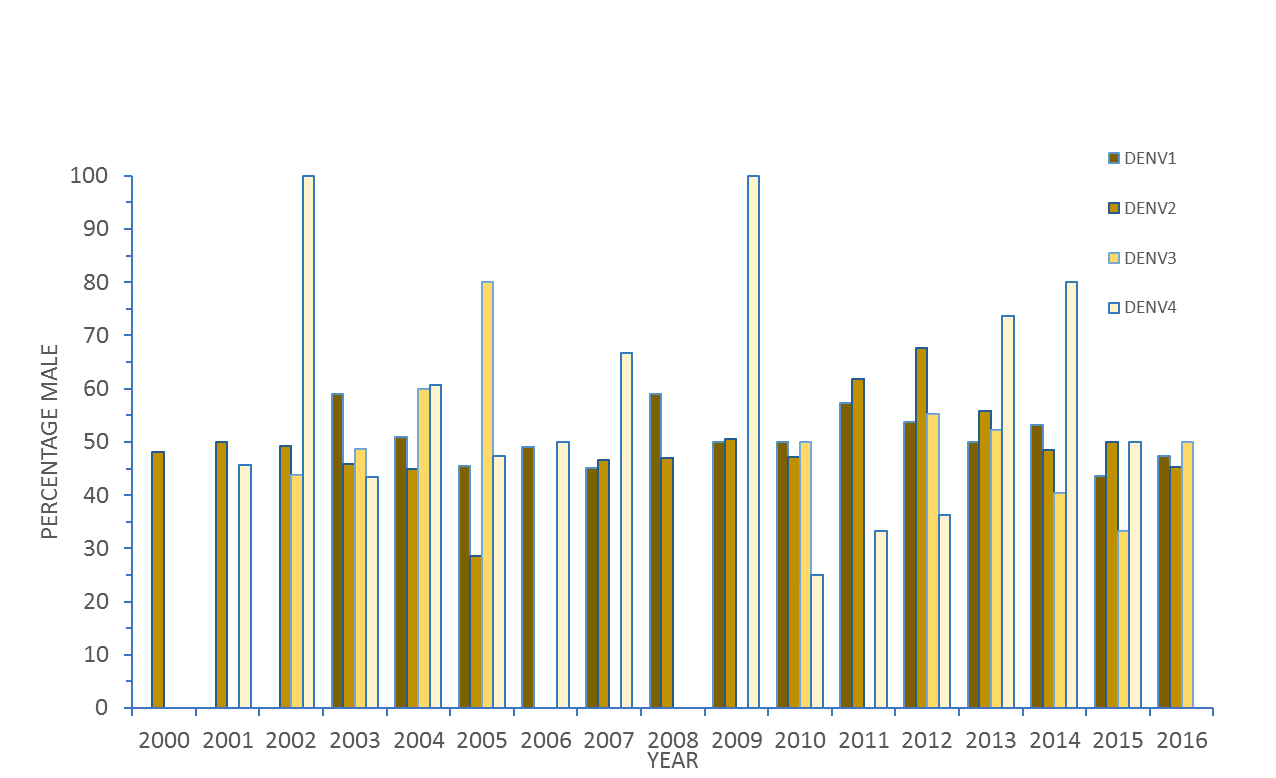

Supplement: S4 Fig — (TIF) [file pntd.0008535.s006.tif]
